# Effects of Motor Skills and Physical Activity Interventions on Motor Development in Children with Autism Spectrum Disorder: A Systematic Review

**DOI:** 10.3390/healthcare13050489

**Published:** 2025-02-24

**Authors:** Yu Xing, Xueping Wu

**Affiliations:** 1 School of Physical Education, Hainan University, Haikou 570228, China; 2 Hainan Provincial Key Laboratory of Sports and Health Promotion, Key Laboratory of Emergency and Trauma Ministry of Education, The First Affiliated Hospital of Hainan Medical University, Hainan Medical University, Haikou 571199, China; 3 School of Physical Education and Training, Shanghai University of Sport, Shanghai 200438, China

**Keywords:** motor intervention, physical activity intervention, autism spectrum disorder, children, motor development

## Abstract

**Background:** Autism spectrum disorder (ASD) is an early childhood and lifelong neurodevelopmental disorder. Many studies have confirmed that motor skills and physical activity interventions can improve motor development in ASD individuals and ultimately improve their quality of life. However, systematic evidence is lacking on whether motor skills and physical activity interventions improve motor development among children with ASD. **Methods:** A systematic search of the CNKI, PsycINFO, PubMed, Web of Science, and Google Scholar databases was conducted for publications through 30 July 2023. Citation tracking and reference tracking were also used, and this study followed the Preferred Reporting Items for Systematic Reviews and Meta-analyses (PRISMA) reporting guidelines. **Results:** Of 8908 studies initially retrieved, 57 met the selection criteria and were evaluated. The overall quality of the evidence, assessed using PEDro, was low. The evaluated studies included 1622 children with ASD, among which 517 were males, from level II to IV, and ranging in age from 3 to 17 years. Five types (physical activity interventions, motor skill interventions, hippotherapy, equine-assisted or simulated horse riding interventions, exergaming interventions, and physical education interventions) of motor development interventions were used, and 57 studies achieved some positive results for improvements in motor development among children with ASD. Furtherly, eight studies reported motor development acquisition, retention, or transfer. Children with ASD learn well from different types of instructors, including teachers, coaches, camp counselors, physical therapists, and peers. **Conclusions:** Motor skills and physical activity interventions improved motor development among children with ASD, the effect of which would continue until the end of the interventions.

## 1. Introduction

Autism spectrum disorder (ASD) is an etiologically heterogeneous and pervasive neurodevelopmental disorder. It often appears in early childhood and is characterized by two core symptoms: repetitive, stereotyped behavior patterns and impairments in social communication and social interaction [1,2]. ASD not only causes patients with difficulties in daily life, including clumsiness, difficulty with voluntary movements, and gross motor coordination disorders but also causes cognitive deficits. Furthermore, the prevalence of ASD increased from 1 in 5000 in 1975 to 1 in 44 in 2021 in the United States, according to the United States Centers for Disease Control and Prevention (CDC); meanwhile, the prevalence of children with ASD aged 0–6 in China was approximately 0.7% in 2022 on the basis of the Specification for Autism Screening and Intervention Services for Children Aged 0–6 reported by National Health Commission [3,4]. The high prevalence of ASD will not only bring great suffering to more people but also impose a huge burden on families and society. Hence, it is practically significant to clarify the interrelation between the external environment and ASD and further to provide physical therapy for children with ASD.

Currently, it is an international consensus that ASD symptoms are correlated with motor developmental disorders in children with ASD, where motor development is a process of increasing coordination and control that occurs throughout the complexity of the lifespan and is closely related to the cognitive, linguistic, emotional, and social development of an individual [5,6]. Many relevant studies have shown that children with ASD have significantly lower motor development than typically developing peers, the gap of which can be easily detected in infancy. Studies also have shown that 59–80% of children with ASD exhibit motor developmental deficits, which is known as the primary symptom of ASD individuals [7]. Related studies have further pointed out that motor developmental disorders may be hidden under the core symptoms of ASD, appearing before the core symptoms [8]. Consequently, deficits in motor coordination, such as gait abnormalities and dysgraphia, have become the focus of research on motor developmental disorders in children with ASD [6]. In this context, researchers propose that motor problems in early child-hood can be used to predict autism and then suggest that motor developmental disorders can be used as one of the early diagnostic indicators for children with ASD [2,7,8]. Therefore, motor developmental deficits not only pose a serious challenge to children with ASD in their daily lives (e.g., dressing, using chopsticks, and drawing) but also induce a range of cognitive and social deficits, leading to a lack of social adjustment in children without intervention. The impairments for children with ASD, including gross motor coordination disorders and cognitive and social deficits, do not alleviate with age but continue into adulthood [8,9]. Thus, motor developmental deficits in children with ASD are considered a part of the overall brain dysfunction in this disorder, the understanding of which may provide new strategies for intervention.

Genetics, neurobiology, and psychology studies have addressed ASD etiology, identification, and intervention [7]. They claim that motor control and coordination deficits in children with ASD are associated with structural abnormalities in the basal ganglia and corpus callosum [7,8]. Gait abnormalities and writing difficulties are mainly related to functional lateralization of the brain as well as to abnormalities in cerebellar circuits [9]. The impaired motor learning ability is primarily associated with the microstructural changes in the cerebellum, and the difficulty in motor execution is closely related to abnormalities of cerebellar activation and cerebral functional networks [10]. The neural basis of sensorimotor dysfunction lies in the abnormalities of the functional connectivity between the visual area and the motor area [11]. Based on these theories mentioned above, a variety of intervention approaches and tools are available for children with ASD, such as applied behavior analysis, structured instruction (e.g., TEACCH), medication, and music therapy. In comparison, these traditional interventions require longer and more intensive cycles, such as >20 h per week for a total of 2 years. Such traditional interventions are usually based on a fixed form of sedentary activity, which limits the options for children with ASD, reduces the amount of time spent in physical activity, and increases the risk of childhood obesity. Motor skills and physical activity interventions show promising prospects for children with ASD due to their high efficiency, good operability, low cost, safety, and lack of adverse effects. Studies by groups such as Rafiei et al. [12], Rifie et al. [13], and Zhou et al. [14] have given some indication of the overall effects of motor development in children with ASD after such interventions. In this case, to help learn these skills, children with ASD may benefit from specific organizational exercises and strategies to support their differences in social communication, behavioral patterns, and interests. However, there is a lack of evidence on the effects of exercise interventions on motor development in children with ASD owing to the large differences between different types of exercise and physical activity interventions.

Motor development refers to the gradual maturation and change in an individual’s motor abilities from birth to adulthood. In contrast, motor skill learning focuses on how an individual improves the performance of specific motor skills through practice and experience. Although some literature reviews [15,16] have reported the effect of motor or physical activity intervention on the motor development of children with ASD, this review differs from previous ones in three aspects, as follows. First, previous reviews mainly focused on the motor outcomes (e.g., body structure, function) of motor skills and physical activity interventions in children with ASD, while this work paid more attention to the motor development of ASD and the influence of localized motor interventions in China such as Taichi and martial arts, trying to propose a motor intervention guideline for children with ASD in a special area. Second, when choosing the implementation environment, previous studies mainly focused on a single one or a simple combination, such as schools, swimming pools, and communities. This review suggests that the diversification of families, schools, and communities is one way to maximize the effectiveness of promoting ASD individuals. Third, unlike previous reviews, this one updated the literature in recent years, and the latest and most valuable studies were included in this work. More importantly, it also found that reliable change index (RCI) values could be used to reflect the individual differences in the motor development of each child, which is very meaningful for future research. Herein, this study aims to systematically review the existing evidence using the International Classification of Functioning, Disability Health for Children and Youth (ICF-CY) framework to assess the methods and progress in motor skills and physical activity intervention therapies for children with ASD. It also explores different types of motor skills and physical activity interventions to provide referential science-based evidence for stakeholders and to broaden the scope of potential interventions for children with ASD. The research hypothesis is that motor skills and physical activity interventions improved motor development among children with ASD, the effect of which would continue until the end of the interventions.

## 2. Materials and Methods

### 2.1. Literature Search Process

This study was reported in accordance with the Preferred Reporting Items for Systematic Review and Meta-Analysis (PRISMA) reporting guideline. The literature was screened mainly through database searches. A search was conducted on China Knowledge Online (1) with the specific search strategy [(AB = “autism” OR AB = “autism spectrum dis-orders”) AND (AB = “motor exercise” OR AB = “sports games” OR AB = “physical activity” OR AB = “sports intervention” OR AB = “sports” OR AB = “exercise”)]; (2) with the subject containing “autism spectrum disorders”, or “ASD”, or “autism” and also “sport”, or “exercise”, or “physical activity”, and “motor/movement development”, or “fine motor”, etc., as specialized search terms on Web of Science (WOS), with the specific search strategy TS = [(“autism spectrum disorder or “ASD” or “autism”) and (“sport” or “exercise” or “physical activity”)]; (3) to collect more relevant studies, with either Chinese or English keywords. The literature search was conducted on CNKI (China National Knowledge Infrastructure), PsycINFO (ProQuest), PubMed (National Library of Medicine), WOS (Web of Science), and Google Scholar. However, some databases (e.g., CNKI) are potentially not being indexed by major search engines such as Google Scholar. For retrieving studies in English citation tracking and reference tracking were also used.

### 2.2. Inclusion Criteria

The literature was screened according to the following five criteria: (1) the study population was children with ASD (≤18 years of age); (2) motor development was the major research goal; (3) this study included an intervention; (4) the intervention was based on motor skills or physical activities; (5) this study investigated the effects of motor skills and physical activity interventions on motor development (acquisition, transfer, or retention). No studies published before 2000 were included because the aim was to use the current definition for the diagnosis of ASD as described in the *Diagnostic and Statistical Manual of Mental Disorders* (Fourth and Fifth Editions) or the Autism Diagnosis Observation Schedule.

### 2.3. Data Extraction and Qualitative Assessment

**Figure 1:**
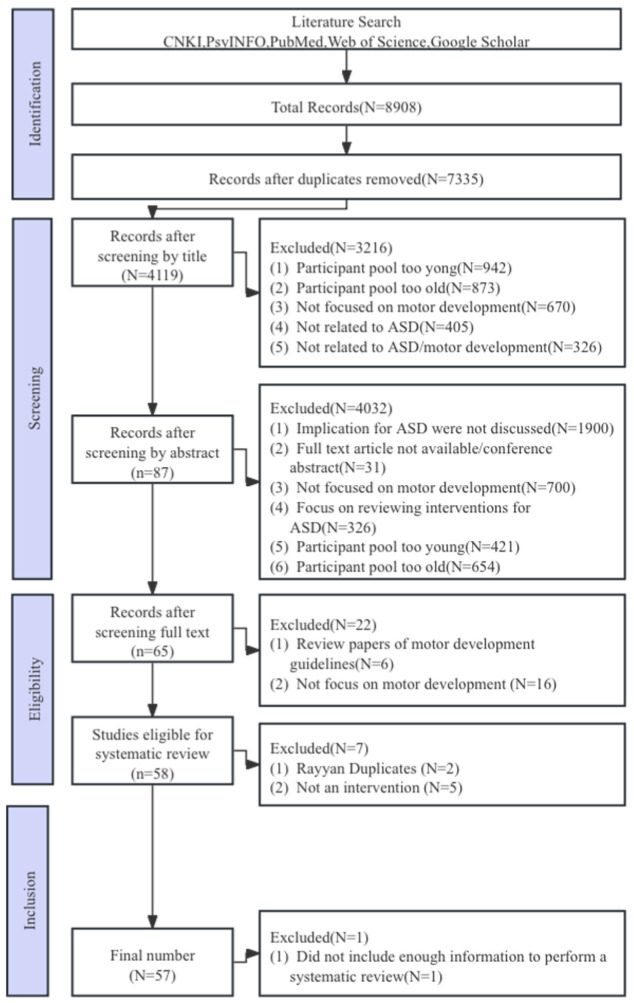
PRISMA flow diagram of the selection of studies.

A total of 8908 studies were retrieved from the aforementioned databases and assessed in RefWorks 6.0 and then in professional version Rayyan to screen for duplicate studies. Data extraction from the remaining studies included the intervention implementation setting, participant characteristics (number, age, and gender), intervention characteristics (content, duration, and periodicity), and assessment methods and effects (immediate effects and maintenance effects), as shown in Figure 1. To ensure the reliability of the results, two experienced researchers independently conducted coding. Methodological quality evaluation of the included studies was conducted based on the Physiotherapy Evidence Database (PEDro), which was independently conducted by two researchers. If there was any disagreement, a third researcher discussed the findings and helped reach an agreement. The inter-rater reliability for all raters was >90%. The quality of studies, summarized in Appendix A, Table A1, was generally low primarily because of a lack of comparison conditions, insufficient power, or small sample sizes.

## 3. Results

### 3.1. Characteristics of Participates

Of the 8908 studies initially identified, 65 articles were reviewed in full text, and finally, 57 articles were included, with a total of 1622 children with ASD, ranging from functional level II to IV, and ages ranging from 3 to 17 years old, including 988 males, 536 females, and 98 unknown genders. Studies published before 2000 were not included in this study, mainly because of the convenience of using the ASD diagnostic definition described in the *Diagnostic and Statistical Manual of Mental Disorders* (4th and 5th editions) or the Autism Diagnostic Observation Schedule.

### 3.2. Design of Research

A total of 20 of the 57 articles adopted a randomized controlled experimental design. Twelve articles grouped the subjects by random grouping using a number list and lottery, and eight articles grouped the subjects by age and degree of disability by stratification. In order to ensure the validity of the intervention, the researchers conducted a homogeneity test on subject demographic data, and four of them used the fact that there was no significant difference in children’s intelligence level, physical level, and motor development level as the starting condition for the intervention.

A total of 22 articles adopted a quasi-experimental design. The researchers fully considered the rigor of randomized controlled experiments, but due to the large individual differences between autistic individuals, scholars compared the intervention effects in the form of changes between groups.

A total of 13 articles adopted a single experimental group design, which paid more attention to the individual characteristics of children with ASD compared with randomized controlled experiments and quasi-experimental designs. In addition, studies have confirmed that “natural growth factors” have little short-term effect on children with ASD, and thus, the intervention effect can be verified by using an unmatched control group and self-control before and after the intervention. In order to further understand the individual effects, Bo proposed to use the credible change index (RCI) to quantify the clinical significance of the changes in each child with ASD before and after the intervention, aiming to solve the clinical differences of children with ASD.

A total of 2 articles adopted a single-subject experimental design. In comparison, the single-subject experimental design can be implemented by considering the basic abilities, personality characteristics, interests, and hobbies of children, which can accurately reflect the motor development trajectory of children with ASD. However, the disadvantage of the small sample size also suggested that future research could integrate groups with ASD under different demographic backgrounds to enhance its promotion value.

### 3.3. Methods of Evolution

The early methods of assessing motor development in children with autism mainly used questionnaires, interviews, and video analysis. The questionnaires and interviews were used primarily to understand the characteristics of the child’s motor development and the expression of idiosyncratic movements through the description of the primary guardian of the child with autism. The video analysis was conducted through an assessment of videos of the child’s early years provided by the parents. In order to better understand the motor developmental characteristics of children with autism, direct testing of the child has become the main form of assessment in recent years. Research of the motor development measures for each study is included, as shown in Table A3. In total, 57 studies used measures such as the *Movement Assessment Battery for Children*, first or second edition (MABC, MABC-2), *Bruininks–Oseretsky Test of Motor Proficiency*, first or second edition (BOT, BOT-2), *Test of Gross Motor Development* second or third edition (TGMD-2, TGMD-3), and Körperkoordinationstest Für Kinder (KTK). Individual measures included goal attainment, such as ball skills, kicking accuracy, traverse speed, and strength. In the following studies, it is becoming important to control for the effects of age, sex, and body mass index on motor development, use objective measures of physical activity, or use medical imaging techniques to represent improvements from interventions objectively. In addition, evaluation can also be performed with targeted measures, such as diaries, logs, and questionnaires. In terms of the effects of exercise interventions, it is important to focus not only on motor development but also on maintenance and transfer effects and to track the immediate and long-term dynamic trajectories of children with ASD.

### 3.4. Characteristics of Intervention: Typology of Exercises/Sports

Due to speech disorders, motor development disorders are particularly prominent in children with ASD during their growth and development stages. Motor development disorders are not only an important sign for early identification of ASD symptoms but also appear to exacerbate the severity of core symptoms such as repetitive, stereotyped behavior patterns and social communication and interaction disorders [13,14,15,16]. Currently, research on motor development in children with ASD has included a variety of interventions, which are no longer limited to jogging. The main exercise interventions shown in Table A2 are discussed below and include the following five categories: (1) physical activity interventions (15 studies: [17,18,19,20,21,22,23,24,25,26,27,28,29,30,31], (2) motor skill interventions (15 studies: [32,33,34,35,36,37,38,39,40,41,42,43,44,45,46]), (3) hippotherapy, equine-assisted, or simulated horse riding interventions (16 studies: [47,48,49,50,51,52,53,54,55,56,57,58,59,60,61,62]); (4) exergaming interventions (6 studies: [12,63,64,65,66,67]); (5) physical education interventions (5 studies: [68,69,70,71,72]). Of 8908 studies initially identified, 65 underwent a full-text review, and 57 met the screening criteria aimed to investigate motor skills and physical activity interventions of motor development among children with ASD.

Unfortunately, few studies have investigated the effects of traditional Chinese sports, such as Tai Chi and martial arts, on the motor development of children with ASD. Taking Tai Chi as an example, as a low-to-medium-intensity aerobic exercise, it has a positive effect on the health of different populations and is believed to have a positive effect on the gait, mood, and cognitive function of middle-aged and older adults with chronic diseases [24,73,74,75]. According to the relevant literature, it can be predicted that patients with ASD can improve their physical coordination ability through regular traditional Tai Chi practice, which is also extremely important for their motor development.

In terms of intervention dose, including time, frequency, and duration, it ranges from 2 to 120 min, 1 to 7 times per week, and lasts for 2 to 48 weeks, but most studies use 10 to 12 weeks of exercise intervention. Unfortunately, there are also great differences in the description of these three dimensions of intervention measures for ASD patients, and few studies have examined the effect size of different types of intervention measures. Fahimeh et al. pointed out that with the increase in intervention time, individuals with ASD improved in both physical fitness and sports participation and appropriately adjusted the intervention time in this study [72,76,77,78]. It can be seen that the current research has not yet reached a consistent conclusion. In the future, it is still necessary to carry out in-depth research and develop scientific exercise intervention guidelines for special populations through repeated verification. This will become an important research direction for special education workers.

### 3.5. Characteristics of Intervention: Place of Implementation

The implementation site of 38 studies was a school, and the intervention implementers included physical education teachers, coaches, summer camp counselors, physical therapists, Tai Chi coaches, and athletes in order to provide professional sports intervention. At the same time, one study used a cooperative model combining family and school, which not only provided ASD individuals with a safe and familiar situation but also provided important value for the acquisition and transfer of intervention effects [42]. In addition, the intervention implementers of the equestrian center were riding coaches, volunteers, occupational therapists, and occupational or physical therapists certified by the Association of Therapeutic Equestrian Professionals and the Italian Federation of Equestrian Sports. It was through a series of activities between the intervention implementers, horses, and participants that the common factors in the environment were subtly increased, which promoted the participants’ understanding of the situational relationship and gradually transferred it to new experience acquisition, thus forming a more stable relationship cognition [52,53]. However, the localization of equine-assisted therapy still needs further exploration. In addition, Pan [57] and Chu [58] proposed that peer support in water training is more likely to promote the motor development of children with ASD, and the implementation of sports intervention for individuals with ASD is diversified.

### 3.6. Effects of Intervention: Acquisition Retention and Migration

A total of 8 studies reported improvements in motor development, retention, and transfer [19,33,35,39,46,49,50], and the results showed that children with weaker social skills made greater progress after motor intervention. Only 1 of the included articles analyzed the RCI values of motor development of children with ASD after intervention. This analysis not only presented the overall development of children with ASD but also systematically analyzed the changes in each patient with ASD [35]. Although the intervention had a positive impact on most patients with ASD, there were a few individuals whose changes were very limited [46]. At the same time, this study also showed that most ASD children lacked appropriate practice opportunities and environments [43]. Future research should consider multi-channel, multi-disciplinary, and multi-faceted research, and for research on children with different degrees of ASD, it is necessary to evaluate their motor development patterns by comparing them with healthy children of the same age so as to develop a systematic intervention plan, promote comprehensive development, and strengthen the test of intervention acquisition, retention, and transfer effects.

Ruggeri et al. [15] and Huang et al. [79] have also published similar studies focusing on the effects of exercise and physical activity interventions on physical fitness, psychological function, and quality of life. The present review assessed the effects of different types of exercise on the motor development of children with ASD. A preliminary exploration was conducted on the environment for intervention implementation, advocating for the localization of traditional Chinese physical activities for the motor development of individuals with ASD. At the same time, attention was paid to the diverse linkage between family, school, and society, enriching the immediate, long-term, and transfer effects after intervention. In addition, the impact of the intervention on each individual with ASD was proposed by scholars such as Bo in recent years, which has important reference value for future research. Future studies should compare the effects of different types of exercise on children with ASD and explore the underlying mechanisms in order to explain the health effects of interventions in this population more comprehensively.

## 4. Discussion

### 4.1. Development of Sensitivity Tool and Local Research

The Denver model suggests that the mechanisms associated with early intervention include utilizing the plasticity of the immature brain to promote complex neural networks and connections through active social engagement, arousal conditioning, and multi-topic, multi-sensory, and multi-domain teaching methods [2]. In the studies evaluated in this review, exercise and physical activity interventions fully mobilized the physical coordination and bilateral limb synergy of children with ASD during the teaching process. Bo et al. believed that the characteristic of exercise intervention is multisensory involvement, and the specific exercise intervention method used in this study (simulated horse riding) may be interpreted by ASD patients as a beneficial external stimulus, thereby helping to improve social motivation and motor development [37]. Most exercise interventions require the joint involvement of the visual, auditory, and proprioceptive cortices, all of which are related to the function of the cerebellum [51]. Therefore, it is reasonable to assume that in the process of exercise intervention, the cerebellum of patients with ASD can be fully stimulated and developed, thereby promoting motor development. Incorporate appropriate sensory stimulation, such as bouncing movements and balance training, into exercise training or clinical rehabilitation to provide crucial sensory input, thereby promoting their motor development and enhancing their social motivation. Conversely, the manifestations of motor development disorders in children with ASD are characterized by externally observable behavioral changes triggered by internal motor nerve impulses under the control of the brain, neural centers, and muscles. Later studies can be combined with assessment tools for the neural mechanisms of motor development or through targeted measures such as diaries, logs, and questionnaires. These can be self-reports, but for children under 10 years old or with lower cognitive function, it is recommended that the primary guardian or teacher fill it out [80]. Both qualitative and quantitative methods have a certain degree of error. Researchers should strive to reduce assessment errors, improve the reliability of their measurement methods and the sensitivity of tools, strengthen local research and development of motor development training materials for children with ASD, carry out pilot work, and continuously improve implementation plans to promote their participation in sports.

### 4.2. Emphasis on Individual Factors in Children and Formulation of Systematic Intervention Plan

For the relevant research on children with ASD of different degrees, it is still necessary to grasp the laws of ASD children’s motor development from the perspective of their motor development, refer to the motor development characteristics of healthy children of the same age, and formulate a systematic intervention plan to promote their all-round development. In view of the fact that most of them show motor development disorders, according to the ICF-CY framework, the comprehensive influence of potential variables such as age, gender, symptom level, intelligence level, and comorbidity factors should be controlled, and sports suitable for the function, motor development and intelligence level of ASD children should be selected. Their sports equipment can also be improved, such as the handrails or safety belts of treadmills, bicycle stabilizing wheels, etc. The choice of exercise method depends on the motor development level and social level of children. For example, children with poor balance ability are more suitable for assisted bicycle training than walking and running. Children with poor coordination prefer to conduct upper and lower limb and whole-body coordination training in a non-competitive environment. In terms of research, the later stage needs to meet statistical requirements and larger sample research, and children with ASD under similar development levels can be used for randomized controlled experiments. In terms of practice, the later stage research still needs to present specific details and focus on the support strategies of different roles. In addition, pay attention to common development and attach importance to individual differences. Group programs may be more suitable for children with milder ASD, while children with more severe ASD may need more individual programs. In general, researchers need to develop systematic intervention plans based on the individual needs of patinates with ASD and make appropriate adjustments to the environment, equipment, content, and rules.

### 4.3. Emphasis of the Joint Drive of Home, School and Community, and Construction of Inclusive Intervention Environment

According to dynamic system theory, the motor learning process is influenced by multiple systems. Therefore, research on sports intervention involves the relationship between individuals, the environment, and motor development [69]. Although most of the interventions reviewed in this article were conducted in schools, rehabilitation centers, and swimming pools, strong family support and social inclusion are also crucial for the development of children with ASD. As mentioned above, children’s motor development depends on the interaction between individuals and the environment [30]. For school-age children with ASD, most of their time is spent in school learning and activities, and family and community are the main places for extracurricular activities. In the context of promoting “Healthy China” and “Sports Power” in China, the joint participation of schools, families and communities is an important implementation path to promote the individual health of children with ASD. Therefore, due to the defects in cognitive and social interaction skills of children with ASD, the intervention site is set in a place they are familiar with while conducting research in unfamiliar places may confuse the results. Later research still needs to emphasize the organic linkage between school, family, and social cooperation. Rehabilitation centers and schools are the main activity places for children with ASD in preschool and school age. In order to facilitate the implementation and promotion of sports intervention courses, physical education teaching reforms can be first carried out in these two places. Although most interventions are based in rehabilitation centers and schools, strong family support and social inclusion are crucial to the development of children with ASD. For following promotion and implementation, it is recommended to encourage parents to cooperate and participate, ensure that the intervention in the family environment is consistent with the school curriculum, and the school can design parent-child activities in the family environment through teaching content and providing corresponding picture books. Families can also actively participate in relevant public welfare activities of social organizations to increase the support of families, schools, and society for children with ASD.

### 4.4. Limitations

The current meta-analysis has several limitations that should be considered when interpreting the results. First, there are a limited number of studies on maintenance and transfer effects, precluding a more comprehensive analysis. Second, there is high heterogeneity among all included studies, which may be attributed to differences in age range, exercise modes, and intervention durations. Additionally, the comparison of intervention effects across different forms lacks further consideration.

## 5. Conclusions

Motor developmental disorders have become an important early predictor of childhood ASD, and understanding the level of motor development in children with ASD is important for their overall development. For improved body-wellness integration, further development of sensitive tools is needed for a study of motor-related mechanisms and early diagnosis of ASD. Intervention programs suitable for children with ASD at different ages and with differing motor abilities are also needed. In addition, there are other important ways to improve the motor development of children with ASD, such as actively attempting traditional Chinese sports as interventions, greatly promoting the diverse linkage between family, school, and society, increasing the immediate and long-term effect evolutions of interventions. These methods can further contribute to the establishment of motor intervention guidelines for ASD individuals, aiming to improve their quality of life. The evaluated studies provided evidence to broaden the scope of exercise interventions for children with ASD, enrich the theory and practice related to motor interventions for children with ASD in China, and offer science-based recommendations for future research and exercise.

## Data Availability

No new data were created or analyzed in this study. Data sharing is not applicable to this article.

## Appendix A

**Table A1:** Evaluation of the level of evidence of all included studies by the Physiotherapy Evidence Database (PEDro) scale.

	Eligibility Criteria Specified	Random Allocation	Concealed Allocation	Baseline Comparability	Blind Subjects	Blind Therapists	Blind Assessors	Adequate Follow-Up	Intention-to-Treat Analysis	Between Group Comparison	Point Estimates and Variability	PEDro Score
Ferguson et al. (2010) [17]	1	0	0	0	0	0	0	1	1	0	1	4
Arzoglou et al. (2013) [18]	1	0	0	1	0	0	0	1	1	1	1	5
Hayward et al. (2016) [19]	1	0	0	1	0	0	0	1	1	1	1	6
Cei et al. (2017) [20]	1	0	0	1	0	0	0	1	0	1	1	5
Guest et al. (2017) [21]	1	0	0	1	0	0	0	1	0	1	1	5
Pan et al. (2017) [22]	1	1	0	1	0	0	0	1	0	1	1	6
Kokaridas et al. (2018) [23]	1	0	0	1	0	0	0	1	1	1	1	6
Sarabzadeh et al. (2019) [24]	1	1	0	1	0	0	0	1	1	1	1	7
Howell et al. (2021) [25]	1	0	0	0	0	0	0	1	1	1	1	5
Ketcheson et al. (2021) [26]	1	0	0	0	0	0	0	1	0	1	1	4
Sansi et al. (2021) [27]	1	1	0	1	0	0	0	1	1	1	1	7
Erisin et al. (2022) [28]	1	1	0	1	0	0	0	1	1	1	1	7
Fahimeh et al. (2022) [29]	1	1	0	1	0	0	0	1	1	1	1	7
Shanker et al. (2022) [30]	1	1	0	1	0	0	0	1	0	1	1	6
Morales et al. (2022) [31]	1	0	0	0	0	0	0	1	1	1	1	5
Cheldavi et al. (2014) [32]	1	0	0	0	0	0	0	1	0	1	1	4
Bremer et al. (2015) [33]	1	0	0	0	0	0	0	1	0	1	1	4
Samsudin et al. (2017) [34]	1	0	0	0	0	0	0	1	0	1	1	4
EI Shemy et al. (2018) [35]	1	1	0	1	0	0	0	1	1	1	1	7
Navaee et al. (2018) [36]	1	0	0	1	0	0	0	1	1	1	0	5
Bo et al. (2019) [37]	1	0	0	1	0	0	0	1	0	1	1	4
Tse et al. (2019) [38]	1	0	1	1	0	0	0	1	1	1	1	6
Tse et al. (2019) [39]	1	0	1	1	0	0	0	1	1	1	0	7
Xing et al. (2020) [40]	1	0	0	1	0	0	0	1	1	1	0	5
Bremer et al. (2021) [41]	1	0	0	1	0	0	0	1	0	1	1	5
Columna et al. (2021) [42]	1	0	1	1	0	0	0	1	1	1	1	7
Dong et al. (2021) [43]	1	0	0	1	0	0	0	1	0	1	0	4
Ketcheson et al. (2021) [44]	1	0	0	1	0	0	0	1	1	0	1	5
Liu et al. (2021) [45]	1	0	1	1	0	0	0	1	0	0	1	5
Bo et al. (2023) [46]	1	0	1	1	0	0	0	1	0	1	1	6
Wuang et al. (2010) [47]	1	0	0	1	0	0	0	1	1	1	1	6
Gabriel et al. (2012) [48]	1	0	1	1	0	0	0	1	1	0	1	6
Ajzenman et al. (2013) [49]	1	0	0	1	0	0	0	1	1	0	1	5
Gabriel et al. (2015) [50]	1	0	1	1	0	0	0	1	0	1	0	5
Srinivasan et al. (2015) [51]	1	0	1	1	0	0	0	1	1	1	1	7
Borgi et al. (2016) [52]	1	0	1	1	0	0	0	1	0	1	0	5
Taheri-Torbati et al. (2018) [53]	1	0	1	1	0	0	0	1	1	1	1	7
Wang et al. (2022) [54]	1	0	0	1	0	0	0	1	1	1	1	6
Pan et al. (2010) [55]	1	0	0	0	1	0	0	1	1	1	1	6
Fragala et al. (2011) [56]	1	0	0	0	1	0	0	1	1	1	1	6
Pan et al. (2011) [57]	1	0	0	0	1	0	0	1	1	1	1	6
Chu et al. (2012) [58]	1	0	0	0	1	0	0	1	1	1	1	6
Alaniz et al. (2017) [59]	1	0	0	0	0	0	0	1	0	1	1	4
Caputo et al. (2018) [60]	1	0	0	0	1	0	0	1	1	1	1	6
Marzouki et al. (2022) [61]	1	0	0	1	0	0	0	1	1	1	1	6
Vodakova et al. (2022) [62]	1	0	0	0	0	0	0	1	1	0	1	4
Hitlon et al. (2014) [63]	1	0	0	0	0	0	0	1	1	0	1	4
Hitlon et al. (2015) [64]	1	0	0	0	0	0	0	1	1	0	1	4
Edward et al. (2017) [65]	1	0	0	1	0	0	0	1	1	1	1	6
Travers et al. (2017) [66]	1	0	0	0	0	0	0	1	1	0	1	4
Caro et al. (2017) [67]	1	0	0	1	0	0	0	1	1	1	1	6
Rafiei et al. (2021) [12]	1	0	0	1	0	0	0	1	1	0	1	5
Sarol et al. (2015) [68]	1	0	0	0	0	0	0	1	1	0	1	4
Henderson et al. (2016) [69]	1	0	0	0	0	0	0	1	1	0	1	4
Toscano et al. (2018) [70]	1	0	0	1	0	0	0	1	0	1	0	4
Chiva-Bartoll et al. (2021) [71]	1	0	0	0	1	0	0	1	0	1	1	4
Fahimeh et al. (2022) [72]	1	0	0	0	1	0	0	1	0	1	0	4

**Table A2:** Study descriptions.

Author(s) (Year)	Study Design, Level of Evidence	Atmosphere	Strength of the Evidence	Participant (Age Range and Diagnosis)	*n* (M)	Intervention Characteristics (Methods/Time/Frequency/Week)	Ratio (Instructor: Child)
(1) **Motor activity intervention**							
Ferguson et al. (2010) [17]	Retrospective cohort, IV	School	Weak	3–8 years, ASD	Total *n* = 3	APA (gross and fine motor skills, endurance, and strength training, bat, ball and sport skill, swinging) 50 min, 3 times/week, 20 weeks	NR
Arzoglou et al. (2013) [18]	Non-randomized trial, III	School	Weak	16 years (mean), ASD	Total *n* = 10, EG:5, CG:5	Traditional Greek dance. 35–45 min, 3 times/week, 8 weeks	1:1–2
Hayward et al. (2016) [19]	Retrospective cohort, IV	Community	Weak	5–19 years, ASD	EG:15	Adaptive soccer program. 90 min, 1 times/week, 6 weeks	1:1
Cei et al. (2017) [20]	Retrospective cohort, IV	School	Weak	6–13 years, ASD	EG:30	Soccer together program. 60 min, 2 times/week, 24 weeks	NR
Guest et al. (2017) [21]	Prospective cohort, IV	School	Weak	6–11 years, ASD	EG:13	Multi-sport camp: locomotor skills and object control skills, translational sports	1:3
Pan et al. (2017) [22]	RCT, II	School	Adequate	6–12 years, ASD	Total *n* = 22 (22), EG:11, CG:11	Table tennis, 40 min, 2 times/week, 12 weeks	1:1–2
Kokaridas et al. (2018) [23]	Prospective cohort, IV	NR	Weak	9 years, ASD and TD	Total *n* = 6 (6), EG:3, CG:3	Indoor climbing, 40 min, 2 times/week, 12 weeks	1:3
Sarabzadeh et al. (2019) [24]	RCT, II	School	Weak	6–12 years, ASD	Total *n* = 18 (14), EG:9, CG:9	Tai Chi Chuan training, 60 min, 3 times/week, 6 weeks	NR
Howell et al. (2021) [25]	Non-randomized trial, II	Community	Weak	5–12 years, ASD and TD	Total *n* = 35, EG:16, CG:19	Football Program(catching, kicking and bouncing), 60–90 min, 1 times/week,13 weeks	NR
Ketcheson et al. (2021) [26]	Non-randomized trial, II	School	Weak	4.67 years (mean), ASD	Total *n* = 25 (18)	Physical Activity Intervention, 60 min, 12 weeks	1:1
Sansi et al. (2021) [27]	RCT, II	School	Adequate	6–11 years, ASD(21)	Total *n* = 45 (34), EG:27, CG:18	Inclusive Physical Activity Program, 60 min, 2 times/week, 12 weeks	1:5
Erisin et al. (2022) [28]	RCT, II	School	Adequate	10.07 years (mean), ASD(14)	Total *n* = 28 (28), EG:14, CG:14	Circuit Exercise Program, 60 min, 3 times/week, 12 weeks	1:1
Fahimeh et al. (2022) [29]	RCT, II	School	Weak	8–11 years, ASD	Total *n* = 28 (28), EG:14, CG:14	ICPL and SPARK, 60 min, 2 times/week, 8 weeks	NR
Shanker et al. (2022) [30]	RCT, II	School	Weak	NR	Total *n* = 43, EG:23, CG:20	Yoga, 45 min, 12 weeks	NR
Morales et al. (2022) [31]	Prospective cohort, III	School	Weak	11.07 years (mean), ASD	Total *n* = 40, EG:21, CG:19	Juda program, 90 min, 1 times/week, 24 weeks	1:5–6
(2) **Motor skill intervention**							
Cheldavi et al. (2014) [32]	Non-randomized trial, III	School	Weak	7–10 years, ASD	Total *n* = 20, EG:10, CG:10	Balance training program, 45 min, 3 times/week, 6 weeks	NR
Bremer et al. (2015) [33]	Non-randomized trial, III	Rehabilitation center	Weak	4 years, ASD	Total *n* = 9 (7), EG:5, CG:4	Fundamental motor skill intervention, 60 min, 1 times/week, 12 weeks; 120 min, 1 times/week, 6 weeks	1:1–2
Samsudin et al. (2017) [34]	Non-randomized trial, III	School	Weak	6–10 years, ASD	Total *n* = 10, EG:5, CG:5	Throwing movements(lobbing) boules program. 10 throws in one set of interventions and a total finished 60 throws in about 2 weeks.	NR
EI Shemy et al. (2018) [35]	RCT, II	School	Weak	8–10 years, ASD	Total *n* = 30, EG:10, CG:10	Gain training with auditory rhythmic cueing, PT program: 60 min, 3 times/week,12 weeks, RAS training: 30 min, 3 times/week, 12 weeks	NR
Navaee et al. (2018) [36]	Non-randomized trial, III	School	Weak	7–10 years, ASD	Total *n* = 20, EG:10, CG:10	A throwing task, 1 x, and retention the next day.	1:1
Bo et al. (2019) [37]	Non-randomized trial, III	School	Weak	8–13 years, ASD	Total *n* = 9	Fundamental movement skills. 60 min, 7 times/week, 2 weeks	1:1
Tse et al. (2019) [38]	RCT, II	School	Adequate	9–12 years, ASD	Total *n* = 65 EG1:22, EG2:22, CG:21	A throwing task, 1 x, and retention/switch the next day.	NR
Tse et al. (2019) [39]	RCI, II	School	Adequate	9–12 years, ASD	Total *n* = 48 EG1:12, EG2:12, EG3:12, EG:12	Basketball shooting task, 6 training blocks, 15 trials, 1 day	1:1
Xing et al. (2020) [40]	RCT, III	Rehabilitation center	Weak	6–10 years, ASD	Total *n* = 24 (18), E:10 (7), C:14 (11).	Fundamental movement skills (run, jump, throwing, catching, and beating), 60 min/times, 3 times/week, 12 weeks	1:2–3
Bremer et al. (2021) [41]	Non-randomized trial, II	NR	Weak	3–5 years, ASD	Total *n* = 20 (15) EG:11, CG:9	Movement skill intervention (running, hopping, catching, jumping, etc.), 60 min, 2 times/week, 12 weeks	NR
Columna et al. (2021) [42]	RCT, III	School, Family	Weak	4–11 years, ASD	Total *n* = 15 EG:8, CG:7	Motor skill intervention (throwing, catching, one-hand strike, etc.), NR	NR
Dong et al. (2021) [43]	Non-randomized trial, III	Rehabilitation center	Weak	6–10 years, ASD	Total *n* = 24 (18) EG:8, CG:7	Fundamental movement skills (run, jump, objective control skills), 60 min, 3 times/week, 12 weeks	1:2–3
Ketcheson et al. (2021) [44]	Non-randomized trial, II	School	Weak	4–6 years, ASD	Total *n* = 20 (15) EG:11, CG:9	Motor Skills Intervenion. 20 min/weeks, 8 weeks	1:1
Liu et al. (2021) [45]	RCT, II	School	Weak	6–10 years, ASD	Total *n* = 24 (24), EG:12, CG:12	Motor intervention (Standing long jump and throwing beanbag), 80 min, 3 times/week, 8 weeks	NR
Bo et al. (2023) [46]	Non-randomized trial, III	Rehabilitation center	Weak	6–10 years, ASD	Total *n* = 24 (18) EG:8, CG:7	Fundamental movement skills (run, jump, objective control skills), 60 min, 3 times/week, 12 weeks	1:2–3
(3) **Hippotherapy, equine-assisted, or simulated horse riding interventions**							
Wuang et al. (2010) [47]	Non-randomized trial, II	Riding therapy	Weak	6–8 years, ASD	Total *n* = 60 (47), EG:30, CG:30	Horse riding intervention, 60 min, 2 times/week, 20 weeks	NR
Gabriel et al. (2012) [48]	Non-randomized trial, II	Riding center	Weak	6–16 years, ASD	Total *n* = 46(36), EG:26, CG:16	Horse riding intervention, 60 min, 1 times/week, 10 weeks	1:3–4
Ajzenman et al. (2013) [49]	Prospective cohort, IV	School-based therapy	Weak	5–12 years, ASD	Total *n* = 6 (4)	Horse riding intervention, 45 min, 1 times/week, 12 weeks	NR
Gabriel et al. (2015) [50]	RCT, II	Riding center	Adequate	6–16 years, ASD	Total *n* = 116, EG:58, CG:58	Horse riding intervention, 60 min, 1 times/week, 10 weeks	1:2–4
Srinivasan et al. (2015) [51]	RCT, II	School	Weak	5–12 years, ASD	Total *n* = 36, EG1:12, EG2:12, CG:12.	Robotic, rhythm, standard-of-care, 45 min, 4 times/week, 8 weeks	1:1
Borgi et al. (2016) [52]	RCT, II	Riding center	Weak	6–12 years, ASD	Total *n* = 28, EG:15, CG:13	Equine-assisted intervention, 60–70 min, 1 times/week, 24 weeks	1:3–4
Taheri-Torbati et al. (2018) [53]	RCT, II	School	Weak	9–13 years, ASD and TD	Total *n* = 48, EG1:12, TD1:1, EG2:12, TD2:12.	Video and live modeling program, 17 training blocks in 2 days, and retention after 1 week	1:1
Wang et al. (2022) [54]	RCT, II	Riding center	Adequate	5–15 years, ASD	Total *n* = 30 (19), EG:15, CG:15	Horse riding intervention, 45–60 min, 2–3 times/week, 24 weeks	1:1
Pan et al. (2010) [55]	Non-randomized trial, III	Swimming pool	Weak	5–9 years, ASD	Total *n* = 16 (16), EG1:8, EG2:8	Aquatic skills, warm-up activities, group games/activities, cool-down activities. 90 min, 2 times/week, 10 weeks	1:2
Fragala et al. (2011) [56]	Non-randomized trial, III	Swimming pool	Weak	6–12 years, ASD	Total *n* = 12, EG:7, CG:5	Aquatic skills, warm-up, aerobic activities, strengthening activities, cool down, and stretching, 40 min, 2 times/week, 14 weeks	NR
Pan et al. (2011) [57]	Non-randomized trial, III	Hydrotherapy and swimming pool	Weak	7–12 years, ASD and TD	Total *n* = 30, ASD:15, TD:15	Aquatic skills, structured social and floor warm-up activities, group games/activities, cool down activities 60 min, 2 times/week, 14 weeks	1:2
Chu et al. (2012) [58]	Non-randomized trial, III	School	Weak	7–12 years, ASD and TD	Total *n* = 42, EG1:14, ASD:7, TD:7, EG2:14, ASD:7, TG:7 EG3:14, ASD:7, TD:7	Aquatic skills with peer-assisted, 60 min, 2 times/week, 12 weeks	1:2
Alaniz et al. (2017) [59]	Prospective cohort IV	Swimming pool	Weak	3–7 years, ASD	Total *n* = 6 (6)	An aquatic therapy program on water safety, 60 min, 1 times/week, 8.16 or 24 weeks	1:2
Caputo et al. (2018) [60]	Non-randomized trial, III	Swimming pool	Weak	6–12 years, ASD	Total *n* = 26, EG:13, CG:13	Aquatic skills, swimming, 45 min, 1–2 times/week, 10 weeks	1:1 1:3
Marzouki et al. (2022) [61]	RCT, II	Swimming pool	Adequate	6–7 years, ASD	Total *n* = 28 (21), EG1:8, EG2:8, CG:6	Aquatic training, technical vs. game-based, 50 min, 2 times/week, 8 weeks	1:2
Vodakova et al. (2022) [62]	Prospective cohort IV	Swimming pool	Weak	9.4 years (mean), ASD	Total *n* = 7 (6)	Aquatic training, swimming, 60 min, 1 times/week, 9 weeks	1:1
(4) **Exergaming interventions**							
Hitlon et al. (2014) [63]	Prospective cohort IV	School	Weak	6–13 years, ASD	Total *n* = 7	Speed-based game (Makotoarena), 2 min, 3 times/week, 10 weeks	NR
Hitlon et al. (2015) [64]	Non-randomized trial, II	School	Weak	8–18 years, ASD	Total *n* = 17	Speed-based game (Makotoarena), 2 min, 3 times/week, 6–10 weeks	NR
Edward et al. (2017) [65]	Non-randomized trial, II	School	Adequate	6–12 years, ASD	Total *n*= 30 (18), ASD:11 (8), TD:19 (10)	AVG, 45–60 min, 3 times/week, 2 weeks	NR
Travers et al. (2017) [66]	Prospective cohort, IV	School	Weak	7–17 years, ASD	Total *n* = 29 (27)	visual-based biofeedback training (Xbox Kinect, Wii), 60 min, 3 times/week, 6 weeks	NR
Caro et al. (2017) [67]	Prospective cohort, IV	School	Weak	7–10 years, ASD	Total *n* = 7	FroggyBobby exergame (Pasitos), 30 min, 2 times/week, 7 weeks	1:2
Rafiei et al. (2021) [12]	RCT, II	School	Adequate	6–10 years, ASD	Total *n* = 60 (16), EG1:20, EG2:20, CG:20	visual-based biofeedback training (Xbox Kinect), 35 min, 3 times/week, 8 weeks	1:2
(5) **Physical education interventions**							
Sarol et al. (2015) [68]	Prospective cohort, IV	School	Weak	4–18 years, ASD	Total *n* = 59	Movement program similar to locomotor, object control and balance, 120 min, 2 times/week, 8 weeks	NR
Henderson et al. (2016) [69]	Prospective cohort, IV	School	Weak	5–12 years, ASD	Total *n* = 37	Locomotor and object control, 40 min, 2 times/week, 20 weeks	1:3
Toscano et al. (2018) [70]	RCT, II	School	Weak	4–18 years, ASD	Total *n* = 64, EG:46, CG:16	Strength, coordination and balance, 40 min, 2 times/week, 48 weeks	1:3
Chiva-Bartoll et al. (2021) [71]	Non-randomized trial, III	School	Weak	10.13 years (mean), ASD	Total *n* = 25 (19), EG:15, CG:10	Physical education program, 60 min, 2 times/week, 28 weeks	1:3–5
Fahimeh et al. (2022) [72]	Non-randomized trial, III	School	Weak	8–12 years, ASD	Total *n* = 30 (17), EG:15, CG:15	Physical literacy program, 80 min, 2 times/week, 8 weeks	NR

Notes: *n*: number, M: male, E: experiment group, C: control group, IQ: Intelligence Quotient; RCT: Randomized Controlled Trial; SPARK: Sport, Play, and Active Recreation for Kids; ICPL: I Can have a physical literacy, CPRT: Class Pivotal Response Treatment, NR: No Reference, I–IV: Level of Evidence.

**Table A3:** Motor development measures for reported studies.

Author(s) (Year)	Assessment Methods	Between-Group Differences Post-Intervention	Within-Group Differences Pre-Post Intervention	Clinical Implications
**Motor activity intervention**				
Ferguson et al. (2010) [17]	MABC	NR	NR	The APA program had a positive effect on improving motor abilities, including improvements in ball skills, manual dexterity, and balance.
Arzoglou et al. (2013) [18]	KTK	NR	↑EG, NS CG	Traditional Greek dance programs improved the agility of children.
Hayward et al. (2016) [19]	Kicking accuracy	NA	↑	The soccer program improved the kicking accuracy and agility of children.
Cei et al. (2017) [20]	Motor skills (walking, running, catching, etc.)	NA	↑	Football training improved motor skills (walking, running, catching, etc.).
Guest et al. (2017) [21]	TGMD-2	NA	↑EG	Multi-sport camp activity improved the locomotor skills and object control skills of girls with ASD.
Pan et al. (2017) [22]	BOT-2	NS	↑EG	Table tennis improved the motor skills of children, compared with a control group, which was maintained for 3 months.
Kokaridas et al. (2018) [23]	Traverse speed Hand grip strength	↑EG(TD) NS	NS NS	Indoor climbing had no difference in ASD between TD.
Sarabzadeh et al. (2019) [24]	MABC-2	↑EG	↑EG, NS CG	Tai Chi Chuan training improved the balance and object control skills of children with ASD, compared with the control group.
Howell et al. (2021) [25]	MABC-2	NS	↑EG, NS, CG	The football program improved in total MABC-2, aiming, catching, and balance, but there is no change in manual dexterity.
Ketcheson et al. (2021) [26]	TGMD-3	↑EG	NR	Physical Activity Intervention improved object control skills.
Sansi et al. (2021) [27]	TGMD-3	↑EG	↑EG, NS, CG	The inclusive physical activity program increased the motor skills of the ASD students and, improved the motor skills of the TD students and positively affected their attitudes towards the ASD students.
Erisin et al. (2022) [28]	BOT-2	↑EG	30%↑EG, CG	Significant improvements in running speed and agility, balance, and standing long jump in EG; only the standing long jump scores failed to improve in CG significantly.
Fahimeh et al. (2022) [29]	BOT	↑EG	↑EG, NS, CG	There were significant differences between EG and CG groups and between the ICPL and Spark programs, which increased MS with children.
Shanker et al. (2022) [30]	BOT-2	↑EG	NR	Yoga programs improve the gross motor rather than fine motor proficiency of children.
Morales et al. (2022) [31]	TGMD-3	↑EG	↑EG, NS, CG	The judo program improves the locomotor skills and gross motor compared with the control group.
**Motor skill interventions**				
Cheldavi et al. (2014) [32]	Postural ability (7 parameters and conditions)	↑EG, CG	NR	Balance training programs improved the postural control of children.
Bremer et al. (2015) [33]	PDSM-2 MABC-2 VABS-II	↑EG, CG NS NS	NR	The fundamental motor skills program improved motor skills with children, compared with the control group.
Samsudin et al. (2017) [34]	Throwing accuracy	↑EG	NR	The throwing movements (lobbing) boules program improved throwing accuracy.
EI Shemy et al. (2018) [35]	BOT-2	↑EG	↑EG, CG	Gaiting training with a rhythmic auditory program improved motor skills in children compared with the control group.
Navaee et al. (2018) [36]	Throwing accuracy	NS	NR	A throwing task did not improve throwing accuracy, and there was no retention compared with the control group.
Bo et al. (2019) [37]	TGMD-3	NA	↑EG	Gross motor skills improve, and the greater the degree of social impairment, the greater the improvement in children’s motor skills.
Tse et al. (2019) [38]	Throwing accuracy	NS	↑EG	A throwing task had no difference between EG1, EG2, and CG; however, there was a retention significance between other groups.
Tse et al. (2019) [39]	Shooting scores	↑EG1, EG2, EG3	↑EG1, EG2, EG3, NS, CG	A basketball task with 4 types improved retention and transfer with children.
Xing et al. (2020) [40]	TGMD-3 MABC-2	↑EG, CG NS	↑EG, NS, CG NS	Fundamental movement skill intervention significantly improved gross motor ability and object control ability.
Bremer et al. (2021) [41]	TGMD-2	↑EG	↑EG, NS, CG	Fundamental movement skill intervention significantly improved their movement skills.
Columna et al. (2021) [42]	TGMD-3	↑EG, CG	EG, NS, CG	Fundamental movement skill intervention significantly improved their movement skills and parents may facilitate the acquisition of skills of their children.
Dong et al. (2021) [43]	TGMD-3	↑EG, CG	↑EG, CG	Fundamental movement skill intervention improved, and the average locomotor skill and object control skills also improved.
Ketcheson et al. (2021) [44]	TGMD-3	NA	↑EG	Fundamental movement skill intervention improved object control skill
Liu et al. (2021) [45]	TGMD-3	NA	↑EG	Fundamental movement skill intervention improved gross motor ability improved the most, followed by displacement motor ability and object control ability improved the least.
Bo et al. (2023) [46]	TGMD-3 MABC-2	↑EG, CG NS	↑EG, NS, CG NS	Fundamental movement skill intervention significantly improved gross motor ability and object control ability.
**Hippotherapy, equine-assisted, or simulated horse-riding interventions**				
Wuang et al. (2010) [47]	BOT-2	↑EG, CG	↑EG, NS, CG	The horse-riding program improved motor proficiency and sensory functions, and the effect was sustained for at least 24 weeks.
Gabriel et al. (2012) [48]	BOT-2	NS	↑	The horse-riding program improved motor skills but did not significance compare with the control group.
Ajzenman et al. (2013) [49]	VABS-II Postural stability (12 COM and COP)	NA	↑EG	The hippotherapy program improved postural stability but did not improve motor skills.
Gabriel et al. (2015) [50]	BOT-2	NS	NR	The hippotherapy program improved motor skills but did not significance compare with the control group.
Srinivasan et al. (2015) [51]	BOT-2	↑CG> EG1, EG2	NS, EG1, EG2, ↑CG	Robotic and rhythmic programs did not improve the manual coordination, body coordination, or praxis of children.
Borgi et al. (2016) [52]	VABS-II	NS	NR	The equine-assisted program did not improve motor skills with children, compared with the control group.
Taheri-Torbati et al. (2018) [53]	NoRMD NoRMS	NS NS	↑EG1, TD1, EG2, TD2 ↑EG1, TD1, EG2, TD2	Video and live modeling programs in similar in children with ASD and TD. There is a similarity in retention between ASD and TD.
Wang et al. (2022) [54]	CP-GMFQ	↑EG	NS	Equine-assisted programs improved motor skills in children but did not have a significant effect compared with the control group.
**Aquatic interventions**				
Pan et al. (2010) [55]	HAAR	↑	↑EG1 ↑EG2	The aquatic skills program improved the swim skills of children, compared with the experiment group, and the effect was sustained for at least 10 weeks.
Fragala et al. (2011) [56]	YMCA Water Skills	NS	↑EG	The aquatic skills program improved the swim skills of children compared with the control group.
Pan et al. (2011) [57]	HAAR	NS	↑EG1, ↑EG2	The aquatic skills program improved the swim skills of children compared with the control group.
Chu et al. (2012) [58]	HAAR	NS	↑EG1, EG2, EG3	Three types of aquatic skills programs did not improve swim skills with children.
Alaniz et al. (2017) [59]	ASC	NA	↑EG	The aquatic skills program improved the swim skills of children.
Caputo et al. (2018) [60]	VABS-II	NS	↑EG, CG	The aquatic skills program improved the swim and motor skills of children.
Marzouki et al. (2022) [61]	TGMD-2	↑EG1, EG2	↑EG1, ↑EG2	The aquatic skills program improved gross motor skills was observed in both experimental groups compared with the control group. No significant between the experimental groups.
Vodakova et al. (2022) [62]	GMFM	NA	↑EG	The aquatic skills program improved the gross motor skills of children.
**Exergaming interventions**				
Hitlon et al. (2014) [63]	BOT-2	NA	NS	Exergaming programs improved the agility of children.
Hitlon et al. (2015) [64]	BOT-2	NA	↑EG	Exergaming programs improved motor performance.
Edward et al. (2017) [65]	TGMD-3	NS	NS	Actual skill scores were not improved in either group. The ASD group improved in perceived skill.
Travers et al. (2017) [66]	BOT-2	NA	↑EG	Biofeedback-based video training programs improved the balance of children.
Caro et al. (2017) [67]	MABC-2	↑EG1, ↑EG2, CG	↑EG1, ↑EG2	Kinect program training improves the motor skills of children compared with the control group. No significant between the experimental groups.
Rafiei et al. (2021) [12]	Limb movements (accurate and simple-aimed)	NA	↑EG	Froggy Bobby program training improves the motor performance of children.
**Physical education interventions**				
Sarol et al. (2015) [68]	PedsQL	NA	↑EG	Movement programs improve the physical functionality of children.
Henderson et al. (2016) [69]	TGMD-2	NA	NS	Locomotor and object control skills improve the motor skills of children.
Toscano et al. (2018) [70]	PH-CHQ	↑EG	NR	Exercise programs improve the physical functionality of children compared with the control group.
Chiva-Bartoll et al. (2021) [71]	MABC-2	NR	↑EG, ↓CG	Physical education program improves manual dexterity manual and balance. However, the results in the control group decreased or remained stable.
Fahimeh et al. (2022) [72]	CAMSA	↑EG	NR	Physical literacy program improves the motor competence of children.

Notes: *n*: number, M: male, E: experiment group, C: control group, IQ: Intelligence Quotient; RCT: Randomized Controlled Trial; SPARK: Sport, Play, and Active Recreation for Kids; ICPL: I Can have a physical literacy, CPRT: Class Pivotal Response Treatment, NR: No Reference, ↑: Increase/Improvement, ↓: Decrease/Deterioration.
